# Socially-parasitic *Myrmica* species (Hymenoptera, Formicidae) of Himalaya, with the description of a new species

**DOI:** 10.3897/zookeys.605.9087

**Published:** 2016-07-14

**Authors:** Himender Bharti, Alexander Radchenko, Sishal Sasi

**Affiliations:** 1 Department of Zoology and Environmental Sciences Punjabi University Patiala; 2 Shmalhausen Institute of Zoology of the National Academy of Sciences of Ukraine, B. Khmelnitsky str., 15, Kiev-30, 01-601 Ukraine

**Keywords:** Ants, Taxonomy, social parasitism, Myrmica latra sp. n., Myrmica ereptrix, Myrmica nefaria

## Abstract

A new socially-parasitic species, *Myrmica latra* **sp. n.** is described based on a queen and male from Indian Himalaya. Its queen differs from other species by the distinctly narrower petiole and postpetiole, blunt and non-divergent propodeal spines, and a darker body colour. The taxonomic position of the three known Himalayan socially-parasitic *Myrmica* species is discussed, and *Myrmica ereptrix* Bolton 1988 is transferred to the *smythiesii* species-group. It is supposed that *Myrmica nefaria* Bharti 2012 is a temporary social parasite, but *Myrmica ereptrix* and *Myrmica latra* **sp. n.** are permanent social parasites, and a key for their identification is provided.

## Introduction

More than 100 years ago, Wheeler (1910) proposed the classification of socially parasitic ants and divided them into four large groups: temporary social parasites, slave-makers, degenerate slave-makers and permanent (or true, workerless = inquilines) social parasites. Basically, a similar classification, but with different terminology was developed by Forel (1922, 1923) (see also Wilson 1971; Buschinger 1986, 1990, 2009, Hölldobler and Wilson 1990).

All socially-parasitic ant species have characteristic morphological features that, taken collectively, were termed as the “inquiline syndrome” by Wilson (1971, 1984) (see also Arnoldi 1930, 1933, Kutter 1973, Bolton 1988, Dowes 1990, Hölldobler and Wilson 1990, Radchenko and Elmes 2003, 2010). The principal features are: reduced size of gynes and males, a widened petiole and especially postpetiole, and the presence of a plate-like tooth or lobe on the ventral surfaces of the petiole and postpetiole. Secondary features for many *Myrmica* social parasites in comparison to free-living species are: much greater body pilosity, spurs on the middle and hind tibiae that are reduced or completely absent, venation in the forewing of alates that is often atypical, and 12-segmented antennae in the males of some species (instead of 13).

The first true socially-parasitic *Myrmica* species, *Myrmica myrmicoxena* Forel, 1895, was discovered in 1869, in a nest of *Myrmica lobicornis* Nylander, 1846, but was not formally described and named until much later, at the end of the 19th century (Forel 1895). A total of 21 species of “true” and putative socially-parasitic *Myrmica* ants have been described from the Holarctic. Some of these species were placed originally in “satellite genera” that have since been synonymised with *Myrmica*; for the taxonomic history of the other generic names see Bolton (1988) and Radchenko and Elmes (2003, 2010). As a result of synonymy only 15 of these names are currently recognized as valid species: eight species from Europe and Algeria, three from North America, two from Siberia and East Asia, and two from the Himalaya (see Radchenko and Elmes 2003, 2010, Francoeur 2007, Bharti 2012, Csösz 2012, Chen et al. 2016).

Recently, the lead author of this paper discovered a queen and a male in Himalaya that possess the typical parasitic *Myrmica* features. Based on differential morphological diagnosis we describe these as a new species *Myrmica latra* sp. n. Additionally, we have compiled a key for the identification of all three known Himalayan socially-parasitic *Myrmica* species.

## Materials and methods

The queen and male of *Myrmica latra* sp. n. were collected by handpicking from nests of *Myrmica aimonissabaudiae* Menozzi, 1939, located under stones. Taxonomic analysis was conducted on a Nikon SMZ 1500 stereo zoom microscope with maximum magnification of 112.5×. For digital images, an MP (Micro Publisher) digital camera was used on the same microscope with AUTO-MONTAGE software (Syncroscopy, Division of Synoptics, Ltd.). Later, images were cleaned with HELICON FILTER 5. The holotype and paratype of new species have been deposited in PUAC (Punjabi University Patiala Ant Collection at Department of Zoology and Environmental Sciences, Punjabi University, Patiala, Punjab, India) and can be uniquely identified with specimen-level codes affixed to each pin (PUAC1569803 and PUAC1569804). Measurements were recorded in millimetres on Nikon SMZ 1500 stereo zoom microscope fitted with ocular micrometer. The comparative morphometric data of the species are listed in Tables 1 and 2.

**Table 1.** Measurements of the Himalayan socially-parasitic *Myrmica* species.

Measurements (in mm)	*Myrmica latra* sp. n.		*Myrmica ereptrix*	*Myrmica nefaria*					
	holotype queen	paratype male	holotype gyne	gynes (n=63)			males (n=4)		
				mean±SD	min	max	mean±SD	min	max
HL	1.23	0.795	1.20	1.13±0.02	1.10	1.17	0.76±0.02	0.74	0.78
HW	1.08	0.63	1.06	1.01 ± 0.01	0.99	1.02	0.69±0.03	0.66	0.71
FW	0.57	--	0.56	0.53 ± 0.01	0.52	0.55	--	--	--
FLW	0.54	--	0.57	0.51 ± 0.02	0.49	0.53	--	--	--
SL	0.90	0.675	0.82	0.87 ± 0.03	0.82	0.92	0.54±0.03	0.50	0.56
PL	0.57	0.40	0.46	0.51 ± 0.02	0.48	0.52	0.39±0.03	0.36	0.42
PW	0.54	0.42	0.65	0.60 ± 0.03	0.58	0.66	0.39±0.02	0.38	0.41
PH	0.54	0.40	0.58	0.54 ± 0.01	0.53	0.54	0.39±0.02	0.38	0.41
PPL	0.48	0.375	0.49	0.45 ± 0.02	0.41	0.49	0.38±0.03	0.36	0.42
PPW	0.87	0.60	0.98	0.95 ± 0.02	0.91	0.97	0.57±0.04	0.53	0.60
PPH	0.84	0.55	0.88	0.81 ± 0.04	0.78	0.89	0.50±0.03	0.47	0.53
ESL	0.21	--	0.19	0.21 ± 0.01	0.19	0.21	--	--	--
ESD	0.48	--	0.56	0.54 ± 0.03	0.46	0.57	--	--	--
AL	2.04	1.47	1.96	1.77 ± 0.02	1.74	1.78	1.35±0.01	1.35	1.36
AH	1.17	0.90	0.96	1.09 ± 0.03	1.06	1.14	0.87±0.005	0.87	0.88
SCW	1.17	0.996	1.06	1.03 ± 0.02	1.00	1.06	0.84±0.03	0.81	0.86
SCL	1.56	1.11	1.54	1.21 ± 0.03	1.14	1.25	0.96±0.03	0.93	0.98

**Table 2.** Morphometric indices of the Himalayan socially-parasitic *Myrmica* species.

Indices	*Myrmica latra* sp. n.		*Myrmica ereptrix*	*Myrmica nefaria*					
	holotype queen	paratype male	holotype gyne	gynes			males		
				mean±SD	min	max	mean±SD	min	max
HL/HW (CI)	1.14	1.26	1.13	1.12±0.02	1.11	1.15	1.10±0.01	1.10	1.12
FW/HW (FI)	0.53	--	0.53	0.53±0.02	0.51	0.54	--	--	--
FLW/FW (FLI)	0.95	--	1.02	0.96 ± 0.05	0.94	1.02	--	--	--
SL/HL (SI_1_)	0.73	0.85	0.68	0.77 ± 0.04	0.75	0.81	0.71±0.06	0.68	0.77
SL/HW (SI_2_)	0.83	1.07	0.77	0.86 ± 0.04	0.83	0.90	0.78±0.02	0.76	0.79
PL/PH (PI_1_)	1.05	1.00	0.79	0.94 ± 0.06	0.91	1.00	0.99 ± 0.14	0.88	1.08
PL/HW (PI_2_)	0.53	0.64	0.43	0.50 ± 0.02	0.48	0.51	0.56 ± 0.02	0.55	0.59
PW/HW (PI_3_)	0.50	0.67	0.61	0.59 ± 0.04	0.58	0.65	0.56 ± 0.02	0.54	0.58
PL/PW (PI_4_)	1.06	0.95	0.71	0.82±0.03	0.77	0.87	0.99±0.10	0.89	1.10
PPL/PPH (PPI_1_)	0.57	0.68	0.56	0.55 ± 0.06	0.49	0.58	0.76 ± 0.11	0.68	0.84
PPH/PPW (PPI_2_)	0.96	0.92	0.90	0.88±0.02	0.86	0.90	0.88 ± 0.00	0.88	0.88
PPW/PW (PPI_3_)	1.61	1.43	1.51	1.52±0.08	1.46	1.68	1.46 ± 0.04	1.40	1.46
PPW/HW (PPI_4_)	0.81	0.95	0.92	0.94 ± 0.04	0.92	0.98	0.82 ± 0.03	0.80	0.85
PPL/PPW (PPI_5_)	0.55	0.63	0.50	0.53±0.02	0.49	0.55	0.67±0.05	0.63	0.72
ESL/HW (ESLI)	0.19	--	0.18	0.21 ± 0.00	0.21	0.21	--	--	--
ESD/ESL (ESDI)	2.20	--	2.95	2.61±0.43	2.38	3.00	--	--	--
AL/AH (AI)	1.74	1.63	2.04	1.78±0.06	1.66	1.83	1.69±0.02	1.67	1.70
SCL/SCW (SCI)	1.33	1.48	1.45	1.01±0.10	0.86	1.11	1.13±0.02	1.12	1.16

**Figures 1.**
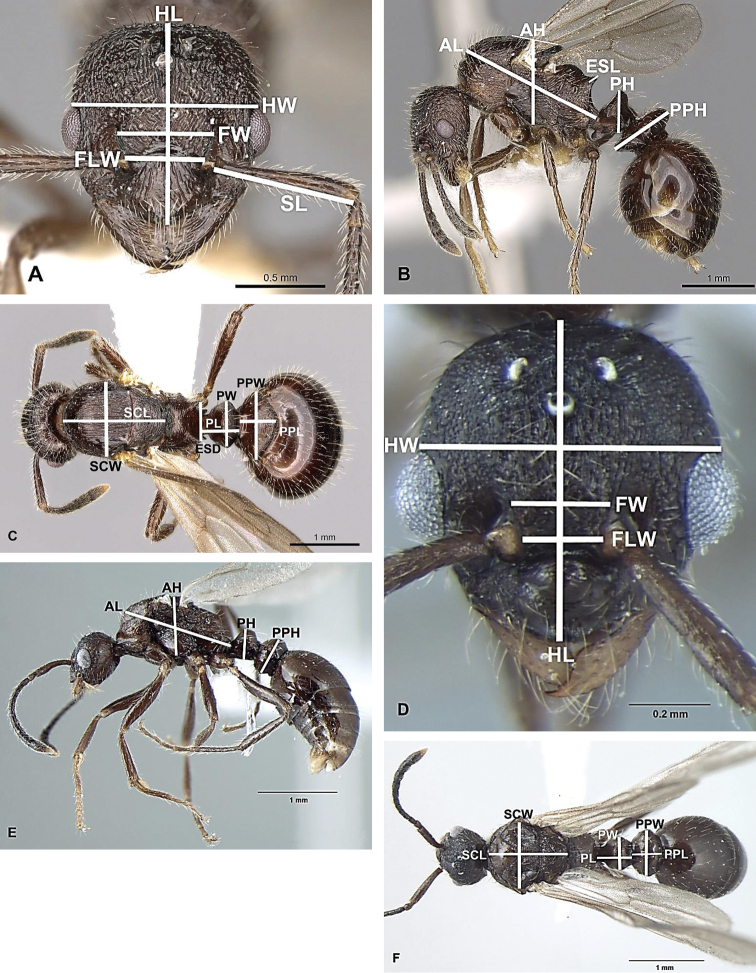
Illustrations: **A** Head (queen) **B** Profile (queen) **C** Dorsum (queen) **D** Head (male) **E** Profile (male) **F** Dorsum (male).

Morphological terminology for measurements (accurate to 0.01 mm) and indices are as follows (see Fig. 1):

HL
 maximum length of head in dorsal view, measured in a straight line from the anterior point of clypeus (including any carina or rugae, if they protrude beyond the anterior margin) to the mid-point of occipital margin 


HW
 maximum width of head in dorsal view behind the eyes 


FW
 minimum width of frons between the frontal carinae 


FLW
 maximum distance between the outer borders of the frontal lobes 


SL
 maximum straight-line length of scape from its apex to the articulation with condylar bulb 


AL
 (= WL-Weber’s length) diagonal length of the alitrunk (=mesosoma) (seen in profile) from the most antero-dorsal point of alitrunk/mesosoma to posterior margin of propodeal lobes 


AH
 height of alitrunk (= mesosoma), measured from upper level of mesonotum perpendicularly to the level of lower margin of mesopleuron in profile view 


PL
 maximum length of petiole in dorsal view, measured from the posterodorsal margin of petiole to the articulation with propodeum; the petiole should be positioned so that measured points lay on the same plane 


PW
 maximum width of petiole in dorsal view 


PH
 maximum height of petiole in profile, measured from the uppermost point of the petiolar node perpendicularly to the imaginary line between the anteroventral (just behind the subpetiolar process) and posteroventral points of petiole 


PPL
 maximum length of postpetiole in dorsal view between its visible anterior and posterior margins 


PPW
 maximum width of postpetiole in dorsal view 


PPH
 maximum height of postpetiole in profile from the uppermost to lowermost point, measured perpendicularly to the tergo-sternal suture 


ESL
 maximum length of propodeal spine in profile, measured along the spine from its tip to the deepest point of the propodeal constriction at the base of the spine 


ESD
 distance between the tips of propodeal spine in dorsal view 


SCW
 maximum width of scutum in dorsal view 


SCL
 length of scutum+scutellum in dorsal view 


### Indices


 Cephalic (CI) = HL/HW


 Frontal (FI) = FW/HW


 Frontal-lobe (FLI) = FLW/FW


 Scape-1 (SI1) = SL/HL


 Scape-2 (SI2) = SL/HW


 Petiolar-1 (PI1) = PL/PH


 Petiolar-2 (PI2) = PL/HW


 Petiolar-3 (PI3) = PW/HW


 Petiolar-4 (PI4) = PL/PW


 Postpetiolar-1 (PPI1) = PPL/PPH


 Postpetiolar-2 (PPI2) = PPH/PPW


 Postpetiolar-3 (PPI3) = PPW/PW


 Postpetiolar-4 (PPI4) = PPW/HW


 Postpetiolar-5 (PPI5) = PPL/PPW


 Propodeal spine-length (ESLI) = ESL/HW


 Propodeal spine-distance (ESDI) = ESD/ESL


 Alitrunk (=mesosomal) (AI) = AL/AH


 Scutum (SCI) = SCL/SCW.

Although the abbreviations of index names have been used in numerous publications (e.g. Radchenko and Elmes 2003) in our experience, many readers find it more convenient to use an explicit description of the ratios, i.e. PPW/PW or PPW/HW instead PPI_3_ or PPI_4_, etc.

## Taxonomy

### Myrmica latra sp. n.

Taxon classificationAnimaliaHymenopteraFormicidae

http://zoobank.org/834B2826-B346-46F2-BA03-5AC2A112D87F

**Figures 2–7.**
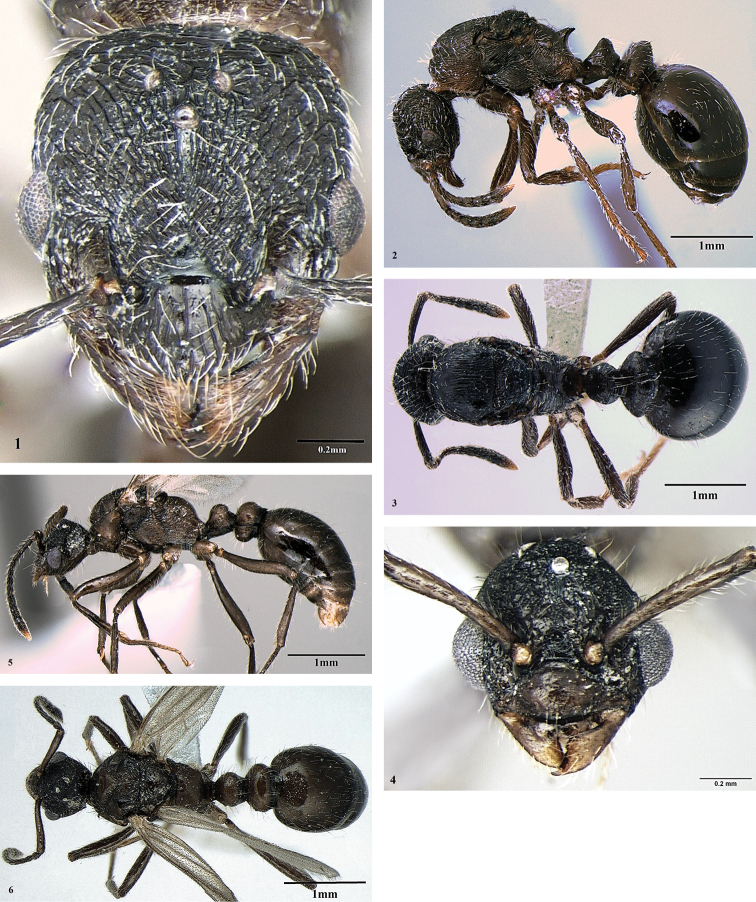
*Myrmica latra* sp. n. **2** Head (queen) **3** Profile (queen) **4** Dorsum (queen) **5** Head (male) **6** Profile (male) **7** Dorsum (male).

Figs 2–7 , Tables 1 , 2

#### Type-material.

***Holotype*** (PUAC1569803) queen, pinned, point-mounted, “India, Himachal Pradesh: Prounthi, 31.1043, 77.6487, 2260m, Hand picking, 14 July 2013, Joginder Singh leg.”. ***Paratype*** (PUAC1569804) male (alate), pinned, point-mounted, “India, Himachal Pradesh, Roggling, 32.5514, 76.9704, 2740m, 12 July 2015, Pawanpreet Kaur leg.” [PUAC]. Nest understone in ground covered with low vegetation and scattered *Pinus* and *Cedrus* trees.

#### Description.

***Queen*** (Figs 2–4, Tables 1–2). Head somewhat longer than broad, with slightly convex sides and occipital margin and widely rounded occipital corners. Anterior clypeal margin convex, but not strongly prominent and not notched medially. Upper latero-ventral corners of head somewhat angulate, but not strongly pointed (seen in profile). Eyes situated slightly in front of midlength of sides of head, Ocelli well developed. Right mandible with 7 teeth, left mandible with 6, apical tooth the largest, preapical one smaller, and other ones uniform and small. Frontal carinae curved outwards to merge with rugae, which surround antennal sockets. Frons wide, frontal lobes converging anteriorly, so that width of frons somewhat wider than distance between frontal lobes. Antennae 12-segmented, with 5-segmented club, scape slender, gradually and feebly curved at the base, without any trace of lobe or carina, shorter than head width, only slightly surpassing occipital margin.

Mesosoma of moderate length, mesonotum feebly convex, scutum not overlapping pronotum, antero-lateral corners of pronotum visible from above, propodeal lobes rounded apically. Propodeal dorsum almost flat (seen in profile). Propodeal spines quite short, widened at the base, thick, not pointed, but narrowly rounded at tips, directed upward (at an angle ca. 45°) and backward, not diverging when seen from above. Metapleural glands moderately large, with conspicuous orifice dorsally on bulla.

Petiole and postpetiole distinctly widened, while less in width in comparison to other Himalayan socially-parasitic *Myrmica* species. Petiole high, with short but distinct peduncle, slightly longer than wide (in other Himalayan socially-parasitic *Myrmica* it is distinctly shorter than wide); its anterior surface concave, node dorsum narrowly rounded; ventral process quite small, widely rounded on tip and directed mostly forward and slightly downward. Postpetiole high, more than 1.5 times higher than petiole, and 1.75 times higher than its length, quite thick and with rather widely rounded dorsum, its anterior surface convex, posterior one almost straight (seen in profile); ventral process well developed, subtriangular, narrowly rounded apically. Spurs of middle and hind tibiae well developed and pectinate.

Head dorsum with coarse longitudinal rugosity and reticulation, diverging postero-laterally. Vertex and occiput with transverse rugosity and reticulation; surface between rugae finely punctate, but appearing shiny. Frontal triangle deep, smooth and shiny. Clypeus longitudinally rugose, surface between rugae finely punctate. Mandibles coarsely longitudinally rugose.

Pronotum longitudinally rugo-reticulate and transverse dorsally. Scutum densely longitudinally rugose, only its anterior part smooth and shiny. Anterior part of scutellum with short longitudinal rugae, its posterior part transversely-concentrically rugose. Propodeal dorsum with finer transverse rugae, its declivity smooth and shiny. Mesopleurae and sides of propodeum longitudinally rugose, only posterior part of anepisternum smooth and shiny. Petiolar node and postpetiolar dorsum transversely rugose. Whole surface of mesosoma between rugae densely while not coarsely punctate, appears dull. Gaster very smooth, polished.

Whole body with whitish hairs. Head dorsum, margins and ventral surface with abundant semi-erect to erect straight whitish hairs of various length, anterior clypeal margin with long setae, mandibles with quite long curved hairs, scape and 7 basal funicular segments with abundant semi-erect to subdecumbent long hairs and shorter pilosity, segments of club with very dense subdecumbent pilosity.

Mesosoma, waist and gaster with numerous long and curved erect hairs, combined with shorter suberect to subdecumbent straight hairs.

Whole body brownish-black, mandibles, antennae, legs (especially tibia and tarsi) and sides of pronotum lighter, brownish.

**Male** (Figs 5–7, Table 1–2). Head distinctly longer than broad, suboval, gradually narrowing behind and in front of eyes, occipital margin convex. Upper latero-ventral corners of head somewhat angulate, but not strongly pointed (seen in profile). Frons somewhat raised up anteriorly and gradually sloping to the level of central ocellus. Clypeus convex, its anterior margin very feebly convex, not prominent and not notched medially. Eyes large in comparison to queen, situated in front of midlength of sides of head, ocelli quite prominent. Mandibles with well-developed apical and smaller preapical teeth, followed by 6 minute blunt denticles. Antennae 13-segmented, with 5-segmented club; scape long, longer than six basal funicular segments and head width, surpassing occipital margin.

Mesosoma long and low, ca. 1.6 times longer than height, scutum and scutellum convex, forming regular arch, scutellum does not project dorsally above scutum when seen in profile. Propodeum gradually rounded, without tubercles, length of its dorsal surface subequal to posterior one, propodeal lobes rounded apically. Petiole with short peduncle, strongly concave anterior surface and widely rounded node dorsum. Postpetiole short and high, ca. 1.5 times higher than length, with evenly rounded dorsum, its sternite looks like a rather long widely rounded ventral plate. Ventral process on petiole small, tooth-like. Both petiole and postpetiole obviously widened. Spurs of middle and hind tibiae well developed and pectinate.

Wing venation almost typical to the genus, e.g. forewing with closed cell *mcu*, open cell *3r*, vein 2+3RS reduced proximally so that cells *1+2r* and *rm* only partly separated.

Head dorsum with irregular short coarse rugae, sides of head and vertex with reticulation. Mandibles smooth, only sparsely punctate, appearing shiny overall. Sides of pronotum mostly smooth, but with fine longitudinal slightly sinuous rugulosity posteriorly. Anterior part of scutum between Mayrian furrows smooth and shiny, its posterior part and scutellum irregularly rugulo-punctate. Anepisternum with irregular fine rugulosity, katepisternum and sides of propodeum coarsely longitudinally rugulose and with fine reticulation; propodeal dorsum and declivity shagreened, somewhat shiny. Petiolar node and postpetiole with fine superficial microsculpture, but appearing more or less shiny. Gaster smooth and shiny.

Whole head surface with numerous long erect to suberect, often curved long hairs and shorter subdecumbent pilosity. Scape and basal funicular segments with subdecumbent to suberect hairs, club segments with subdecumbent short pubescence. Mesosoma and waist with abundant, quite long suberect to erect hairs, gaster with similar long hairs and sparse short subdecumbent pilosity. Legs with numerous subdecumbent, quite long hairs. Whole body and appendages brownish.

**Workers.** unknown.

#### Remarks.

The queen of *Myrmica latra* sp. n. differs from the known non-parasitic Himalayan *Myrmica* species by possessing characteristic features of the “inquiline syndrome”, particularly by the distinctly widened petiole and postpetiole, presence of the well-developed ventral lobe on the petiole and postpetiole, and also by the presence of more hair on the body. Although *Myrmica latra* shares these features with two already described socially-parasitic Himalayan species, *Myrmica ereptrix* Bolton, 1988 and *Myrmica nefaria* Bharti, 2012, it differs from both by in following characters: *Myrmica latra* has a relatively less-widened petiole and postpetiole, its head is twice as wide as the petiole: PW/HW = 0.50 compared to PW/HW = 0.58-0.65 in the two other species; PPW/HW = 0.81 in *Myrmica latra* versus a ratio > 0.92 in the other two species. The petiole in *Myrmica latra* sp. n. is nearly as long as wide (PL/PW = 1.06), but in the other two it is distinctly wider than long (PL/PW ≤ 0.85); the ratios PPL/PPW are 0.55 *vs.* ≤ 0.55, respectively. Other differences include the ventral processes on the petiole and postpetiole in *Myrmica latra* being distinctly smaller than in *Myrmica ereptrix* (compare Figs 3 and 9); its propodeal spines are blunt and not divergent, while in both *Myrmica ereptrix* and *Myrmica nefaria* they are pointed and distinctly divergent (compare Figs 3 and 9, 12); the spur on the middle tibiae in *Myrmica ereptrix* is strongly reduced, while in the other species it is well developed and pectinate; the body colour of *Myrmica latra* sp. n. is darker than in two other species.

**Figures 8–10.**
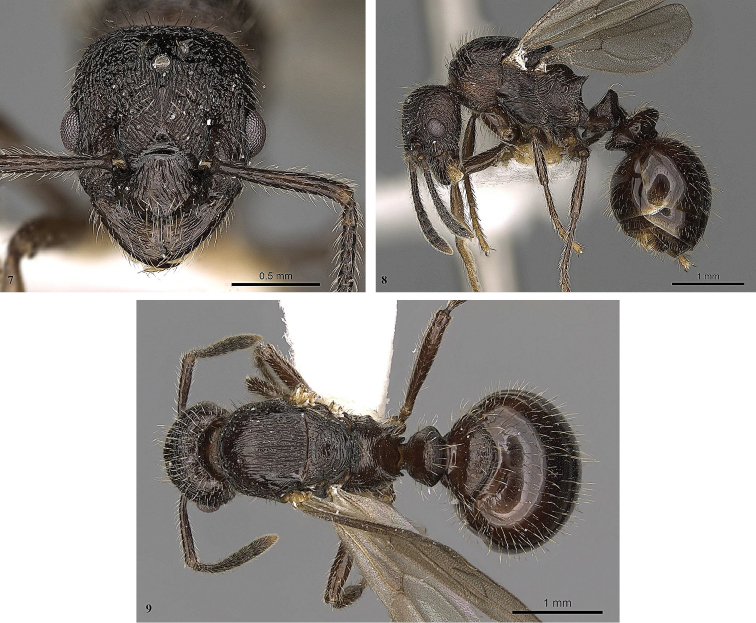
*Myrmica ereptrix*. **8** Head (queen) **9** Profile (queen) **10** Dorsum (queen).

**Figures 11–16.**
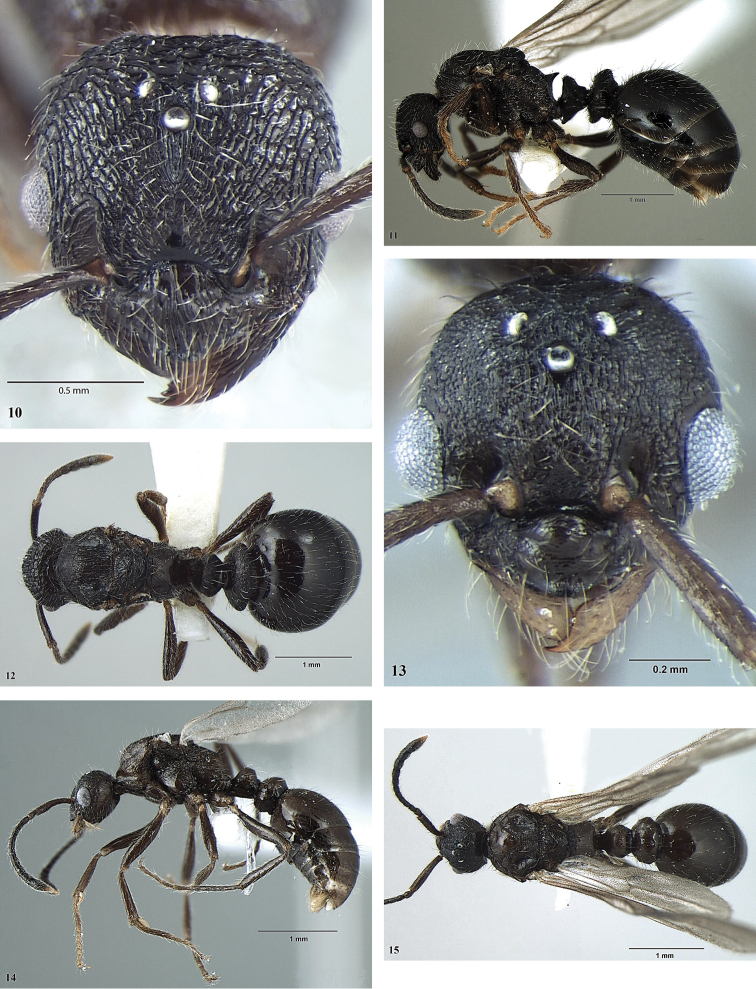
*Myrmica nefaria*. **11** Head (queen) **12** Profile (queen) **13** Dorsum (queen) **14** Head (male) **15** Profile (male) **16** Dorsum (male).

The male of *Myrmica latra* sp. n. well differs from all the known males of the species of the *smythiesii*-group (see also Discussion, below) by the much wider petiole and postpetiole, as well as by the distinctly higher postpetiole, its sternite gives the appearance of rather long and widely rounded ventral plate. Thus, in *Myrmica latra* PW/HW = 0.67, PPW/HW = 0.95 and PPL/PPH = 0.68, but these ratios in the non-parasitic species from the *smythiesii*-group (*Myrmica bactriana* Ruzsky, 1915, *Myrmica fortior* Forel, 1904 and *Myrmica ruzskyana* Radchenko et Elmes, 2010) are: PW/HW < 0.40, PPW/HW < 0.60 and PPL/PPH > 0.80 (our unpublished data).

While the male of *Myrmica latra* morphologically resembles the male of *Myrmica nefaria* (the males of *Myrmica ereptrix* are unknown), it differs by its longer head (HL/HW = 1.26 *vs.* 1.10–1.12) that is distinctly narrowed posteriorly (compare Figs 5 and 14); by the distinctly longer scape that is longer than the head width in *Myrmica latra*: SL/HL = 0.85, SL/HW = 1.07 *vs.* SL/HL = 0.68–0.77 and SL/HW = 0.76–0.79; by the wider petiole and postpetiole (PW/HW = 0.67, PPW/HW = 0.95 *v*s. 0.54–0.58 and 0.80–0.85). Additionally, the head dorsum in *Myrmica latra* has short irregular rugae, but in *Myrmica nefaria* males, the head dorsum has longitudinal rugae; posterior part of scutum has longitudinal rugae *vs.* transversal rugosity; its propodeum is gradually rounded, without teeth or tubercles, but in *Myrmica nefaria* propodeum is distinctly angulated with short teeth. Finally, the forewing venation of the male of *Myrmica latra* sp. n. is almost typical for the genus *Myrmica* and resembles that of *Myrmica ereptrix* (see above and Bolton 1988), but in some males of *Myrmica nefaria* it is modified (see Bharti 2012). However, it should be remembered that the forewing venation in different specimens of the same species, especially in social parasites, may be quite variable so not too much reliance should be placed on this feature (see Arnoldi 1930, 1933; Bolton 1988; our own observations).

#### Etymology.

From the Latin adjective *latra*, meaning robber or thief.

#### Ecology.

Both queen and male were collected from nests of *Myrmica aimonissabaudiae* built under stones. The ground is covered with low vegetation, and scattered *Pinus* and *Cedrus* trees. The recorded nest temperature and humidity at site one, where queen was collected was 18 °C and 76%, whereas at site two, where male was collected, the recorded nest temperature was 19 °C and humidity 66%.

### Key for identification of the socially-parasitic Himalayan *Myrmica* species

**Queens**

**Table:** 

1	Petiole and postpetiole narrower, PW/HW = 0.50, PPW/HW = 0.81, petiole nearly as long as wide, PL/PW = 1.06 (Figs 3, 4). Propodeal spines blunt and not divergent (Figs 2–4). Body colour darker, blackish-brown	***Myrmica latra* sp. n.**
–	Petiole and postpetiole wider, PW/HW = 0.58-0.65, PPW/HW > 0.90, petiole distinctly wider than length, PL/PW ≤ 0.87 (Figs 9, 10, 12, 13). Propodeal spines pointed and distinctly divergent (Figs 10, 12). Body colour lighter, reddish-brown	**2**
2	Head dorsum with longitudinal rugae and reticulation (Fig. 11). Dorsal surface of propodeum with divergent longitudinal rugae (Fig. 13). Middle and hind tibiae with well-developed pectinate spur (Fig. 12). Petiole somewhat longer, PL/PH > 0.90, PL/PW = 0.77–0.87; mesosoma relatively higher, AL/AH =1.66–1.83	***Myrmica nefaria* Bharti**
–	Head dorsum with longitudinal, somewhat divergent rugae, reticulation present only on vertex and temples (Fig. 8). Dorsal surface of propodeum transversally rugose (Fig. 10). Hind tibiae with well-developed pectinate spur, but spur on middle tibiae strongly reduced, short and simple (Fig. 9). Petiole somewhat shorter, PL/PH = 0.79, PL/PW = 0.71; mesosoma relatively lower, AL/AH =2.04	***Myrmica ereptrix* Bolton**

**Males** (males of *Myrmica ereptrix* are unknown)

**Table:** 

1	Head longer, HL/HW = 1.26, distinctly narrowed posteriorly above eyes; head dorsum with short irregular rugae (Fig. 5). Scape longer, SL/HL = 0.85, SL/HW = 1.07. Petiole and postpetiole wider, PW/HW = 0.67, PPW/HW = 0.95; posterior part of scutum with longitudinal rugae (Fig. 7). Propodeum gradually rounded, without teeth or tubercles (Fig. 6)	***Myrmica latra* sp. n.**
–	Head shorter, HL/HW = 1.10–1.12, gradually arched above eyes; head dorsum with longitudinal rugae (Fig. 14). Scape shorter, SL/HL = 0.68–0.77, SL/HW = 0.76–0.79. Petiole and postpetiole narrower, PW/HW = 0.54–0.58, PPW/HW = 0.80-0.85; posterior part of scutum with transversal rugosity (Fig. 16). Propodeum distinctly angulated and with short teeth (Fig. 15)	***Myrmica nefaria* Bharti**

## Discussion

There are two questions that need to be addressed: first, why have we described this queen and male that were collected from different nests as the same species? Secondly, why have we described them as social parasites?

The second question is more easily answered: both castes possess a combination of features known as the “inquiline syndrome” (discussed above) and by these features they significantly differ from all known free-living Himalayan *Myrmica* species. This species is most unlikely to occur elsewhere, given that the *Myrmica* fauna of the Himalayan region is almost completely isolated from the fauna of adjacent regions (Radchenko and Elmes 2001, 2010). If *Myrmica latra* is a social parasite then the queen well differs from those of the two known Himalayan socially-parasitic species, *Myrmica ereptrix* and *Myrmica nefaria*, while the male differs significantly from those of *Myrmica nefaria* (males of *Myrmica ereptrix* are unknown).

**Map 1.**
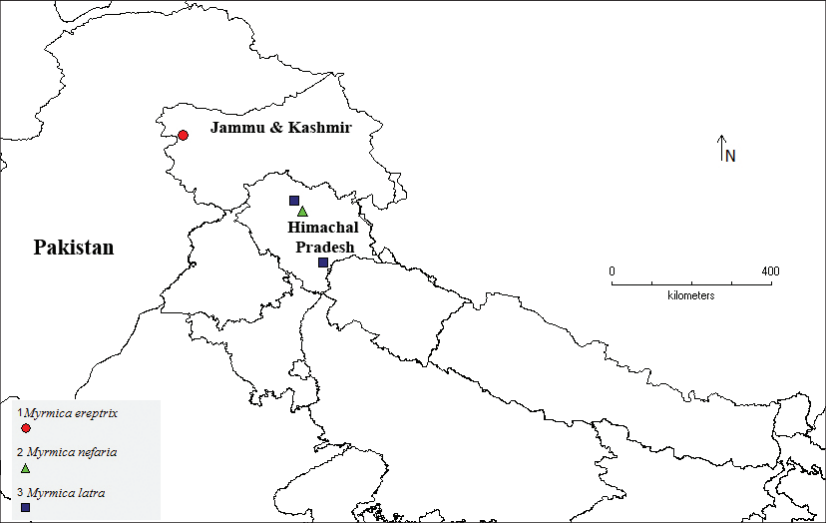
Geographical distribution of socially parasitic species in Himalaya.

We have decided to describe the queen and male as the same species, despite coming from different nests, because the putative host colonies were of the same species, *Myrmica aimonissabaudiae*, living in the same general region at similar altitudes albeit the two sites were 173 km apart (see Map 1). *Myrmica aimonissabaudiae* is now known to host two socially parasitic species (*Myrmica ereptrix* and *Myrmica latra*) and while there is no reason why it should not host several more species (e.g. as in the case of *Myrmica sabuleti* Meinert in Europe) the simplest hypothesis this queen and male belong to the same species. With our present knowledge, we do not wish to create an extra name, which might be synonymised later.

Furthermore, it is always better to avoid the description of a new taxon based on a single specimen, especially, if it is collected in isolation (e.g. in a pitfall trap), but in this case the specimens were collected from a nest of same host species and both male and female differ from already known species of the genus. To date, eight *Myrmica* species have been described based on a single queen (Emery 1907, Bernard 1967, Bolton 1988, Radchenko and Elmes 1999) or worker (Forel 1902, Radchenko et al. 2008, Radchenko and Elmes 2009), but no valid *Myrmica* species have been described based on males. Therefore, we have designated the queen as the holotype and male as a paratype. If, in the future, queens of *Myrmica latra* are found with males in the same host nest (or collected *in copula* in a mating swarm) and the males are distinctly different from the paratype male of *Myrmica latra* described here, then the specimen in question would be validated as a separate species. Additionally, it is quite logical to designate the queen as holotype, as male-based taxonomy in the genus *Myrmica* is much less developed than the female-based one, and in many cases correct identification of a single male is nearly impossible (see Radchenko and Elmes 2010, Czechowski et al. 2012).

The present concept of species-groups in the genus which is based on morphology, was outlined by Radchenko (1994) and further improved by Radchenko and Elmes (2001, 2010), and currently in the absence of a complete molecular phylogeny, this concept is quite useful to indicate the degree of relatedness between species. Although, a molecular phylogeny based on a sample of *Myrmica* species (Jansen et al. 2010) mostly complemented the morphological species-group concept (Radchenko and Elmes 2010). However, in the light of present findings, we ought to reconsider the *rugosa* and *smythiesii* species groups.

The molecular genetic analysis published by Jansen et al. (2010) did not support the separation of the *rugosa* and *smythiesii* species groups. In the above mentioned analysis, three of the *rugosa*-group species were analyzed (*Myrmica rugosa* Mayr, 1865, *Myrmica aimonissabaudiae* Menozzi, 1939, and *Myrmica rupestris* Forel, 1902) along with *Myrmica wittmeri* Radchenko et Elmes, 1999 (a quite peculiar species in some characters that was tentatively placed in the *smythiesii*-group). Besides, the material of “*Myrmica rugosa*” was collected in Kyrgyzstan, well outside the limits of known geographic distribution of this species, either this was a typing error in the paper or the specimens were misidentified. Moreover, in the above cited phylogenetic analysis, the American *Myrmica wheeleri* Weber, 1939 (that quite well differs morphologically from the Himalayan species) is grouped with the species of “*rugosa*-group”. Thus, these intriguing results indicate that there are still a lot of taxonomic problems within the supra-specific taxonomy of the Himalayan *Myrmica*, until a molecular analysis with inclusion of many more species is carried out; the morphological species-groups still have some usefulness.

Morphologically, female castes of the *rugosa* and *smythiesii* groups share several diagnostic features (e.g.: scape very smoothly curved at the base, not angled and with no trace of a lobe or carina; frontal lobes slightly curved, frons wide and frontal lobes not extended; anterior clypeal margin is convex and prominent, without a medial notch). The main difference is the shape of the frontal carinae: in the *rugosa*-group they merge with the rugae that extend to the occipital margin, do not curve outwards and do not merge with rugae that surround antennal sockets, but in the *smythiesii*-group frontal carinae curve outwards to merge with the rugae that surround the antennal sockets. In addition, males of the *rugosa*-group have a relatively short scape, SL/HL < 0.60, but those of the *smythiesii*-group have much longer scape – SL/HL > 0.70 but unfortunately males are unknown for some species in this group: *Myrmica wittmeri*, *Myrmica bactriana* Ruzsky and *Myrmica ruzskyana* Radchenko & Elmes. If the *rugosa*- and *smythiesii*- species groups are quite closely related, then, taking into account the length of scape in males, species placed in the latter group are obviously more evolved, because a short scape is a plesiomorphic state not only for *Myrmica*, but for ants as a whole (see Radchenko and Elmes 2001, 2010, Radchenko et al. 2007, Dlussky and Radchenko 2009).

Regarding the Himalayan social parasites: when Bolton (1988) described *Myrmica ereptrix* from a single gyne found in the nest of *Myrmica aimonissabaudiae*, the present species-group concept in the genus *Myrmica* was not fully established, and he placed this species in the *rugosa*-group. Later, Radchenko and Elmes (2001, 2003, 2010) erroneously subscribed to his viewpoint. However, based on the diagnostic features of species-groups (discussed above), now we formally transfer *Myrmica ereptrix* to the *smythiesii*-group (*Myrmica nefaria* and *Myrmica latra* are also placed in this group, see also Bharti 2012), while the host species of *Myrmica ereptrix* and of *Myrmica nefaria* belong to the *rugosa*-group. Generally, the social parasites of *Myrmica* are phylogenetically close to their hosts (Jansen et al. 2010) and we may only suppose that these parasites evolved at the same time when the *smythiesii*-group was diverging from the *rugosa*-group (Bharti 2012).

Probably, *Myrmica nefaria* is a temporary social parasite as all its castes were found in the host colony and in the right circumstances may potentially form free-living colonies (as in the case of *Myrmica vandeli* Bondroit, 1920 in Europe (see Elmes et al. 2003, Radchenko and Elmes 2003, 2010). At the moment, we can only speculate on the life-style of the other two species, most probably they are obligate social parasites.

## Supplementary Material

XML Treatment for Myrmica latra
